# Learning Arm/Hand Coordination with an Altered Visual Input

**DOI:** 10.1155/2010/520781

**Published:** 2010-07-19

**Authors:** Simona Denisia Iftime Nielsen, Strahinja Došen, Mirjana B. Popović, Dejan B. Popović

**Affiliations:** ^1^Center for Sensory-Motor Interaction (SMI), Department of Health Science and Technology (HST), Aalborg University (AAU), DK-9220 Aalborg, Denmark; ^2^Faculty of Electrical Engineering, University of Belgrade, Belgrade 11120, Serbia; ^3^Institute for Multidisciplinary Research, Belgrade 11030, Serbia

## Abstract

The focus of this study was to test a novel tool for the analysis of motor coordination with an altered visual input. The altered visual input was created using special glasses that presented the view as recorded by a video camera placed at various positions around the subject. The camera was positioned at a frontal (F), lateral (L), or top (T) position with respect to the subject. We studied the differences between the arm-end (wrist) trajectories while grasping an object between altered vision (F, L, and T conditions) and normal vision (N) in ten subjects. The outcome measures from the analysis were the trajectory errors, the movement parameters, and the time of execution. We found substantial trajectory errors and an increased execution time at the baseline of the study. We also found that trajectory errors decreased in all conditions after three days of practice with the altered vision in the F condition only for 20 minutes per day, suggesting that recalibration of the visual systems occurred relatively quickly. These results indicate that this recalibration occurs via movement training in an altered condition. The results also suggest that recalibration is more difficult to achieve for altered vision in the F and L conditions compared to the T condition. This study has direct implications on the design of new rehabilitation systems.

## 1. Introduction

Visual information plays an important role in both planning and executing goal-directed movements. When planning the *reaching* aspect of the *“reach to grasp movement,”* vision provides information about the object's properties (shape, size, and position in space) as described in detail many years ago by Jeannerod [1]. During the execution of the action, the proprioceptive system (muscle spindles, Golgi tendon organs, and joint receptors) sends information to the central nervous system, which is then used for estimation of the accuracy of the execution. In parallel, vision provides feedback, which allows corrections if they are required [2]. The performance depends on the level of mastery in executing the movement that follows the learning. 

The role of vision during reaching to grasp was studied in detail by either preventing the subject from viewing either only the hand or both the object and the hand during movement (this is often referred to as visual open loop; e.g., [3–5]). The results of previous studies agree that preventing vision during the reaching movement affects movement parameters (i.e., hand-target distance at the initiation of aperture closure, grip aperture amplitude, wrist velocity, and acceleration) and the relationship between those parameters. Movement time tends to increase when visual feedback is impaired, mostly due to a longer deceleration phase of the movement caused by a slower approach to the object [5–9]. This increase in movement time was found when visual feedback was blocked during the entire movement, and not when this feedback was only blocked during the initial part of the movement [5, 9–11], when vision of the hand was blocked [5–7, 12, 13], and when monocular vision was used [8, 14, 15]. 

The brain can adapt to a variety of distortions of visual feedback when reaching for targets, including rotations and lateral shifts, by adjusting hand movements [16, 17]. This adjusted hand movement can be retained in all subjects after 24 hours [18] or even a year later [17]. The novel dynamic environment learned for a single movement can be generalized to movements of the same orientation of either increased rate or amplitude [19]. 

Cerebrovascular accident (i.e., stroke) often results with paralysis (decreased or complete loss of abilities to manipulate the grasp), but also leading to modified association of proprioceptive and visual information coming to the brain and preventing the brain from sending necessary command signals to the periphery [20, 21]. Therefore, there is a need for stroke patients to relearn how to integrate the preserved mechanisms into a functional reach to grasp movement. This was the motivation to study the learning of new motor coordination skill using dissociated visual and proprioception systems. 

This paper presents the analysis of how one learns to make hand movements in a new visuoperceptual association generated by a simple tool for altering visual input. The alteration of visual input was achieved with commercially available computer goggles (Myvu Crystal EV, http://www.myvu.com/) developed for the iPod. The goggles integrate two miniature video monitors into the left and right eye covers. We connected the goggles to the video output of a high-resolution digital camera. Thus, the visual input to the subjects was the image seen by the digital camera. 

We analyzed the learning outcome when vision was altered by presenting the scene recorded by the camera placed at three locations around the working space. The analysis of movement errors relates only to the *reaching* part of the *reach and grasp* task. The task analyzed was a “reach and grasp a small object”. The execution of the task was grossly divided into successful (objects grasped) and nonsuccessful (object missed). We analyzed the performance on the day one and on day five allowing subjects to practice for three consecutive days with the goggles. This research follows studies related to the so-called perceptual recalibration that takes place when the subject is exposed to altered visual input. It was suggested that when a discrepancy was introduced between the “seen” and “felt” location of an object [22], performance suffered. However, the sensory systems rapidly adapt to this discrepancy, returning perception and performance to near normal. Interestingly, subsequent removal of the discrepancy leads to a decrease in performance, known as the so-called Negative Aftereffect [23]. One of the suggestions is that this adaptation consists of “recalibrating” the transformation between the visual and proprioceptive perception of spatial location [24] because visuomotor adaptation is a perceptual recalibration that depends on the subject's familiarity with the trajectory [23].

## 2. Methods

### 2.1. Subjects

Ten healthy volunteers (mean age: 26 ± 4 years; range 25–35 years) with no history of neuromuscular or visual disorders participated in the experiment. All subjects signed the informed consent prior to the experimental sessions. The investigation complied with the declaration of Helsinki and was approved by the Local Ethics Committee.

### 2.2. Procedure

**Figure 1:**
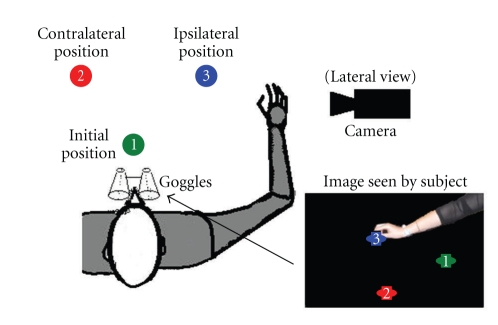
The sketch of the setup. The right bottom insert shows the image displayed to the subject (goggle view).

The subjects sat comfortably in a chair in front of a standard large desk covered with a black cloth (Figure 1). The trunk was fixed by a belt to the back support of the chair to minimize the motion of the shoulder during the reach to grasp tasks. The height of the chair was adjusted to allow for motion of the hand just above the table surface. The experiments were performed with the right (dominant) hand only. At rest (initial position of the hand), the elbow was flexed at about 60° and the shoulder at about 30°. Three colored circles with an 8 cm diameter were fixed to the cloth within the workspace; these represented the initial hand position (green—1), contralateral target (red—2), and ipsilateral target (blue—3). Distances between the circles were adjusted individually for each subject, so that subjects could comfortably reach them without fully extending their elbow. The range of distances was 35 to 50 cm. 

**Figure 2:**
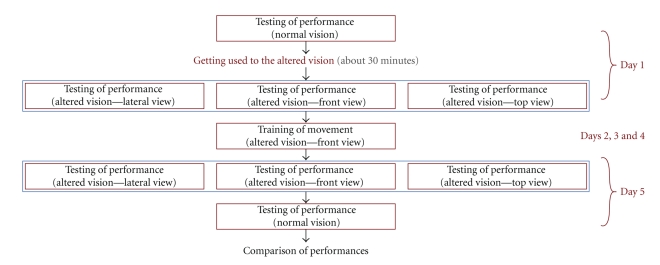
Schematic presentation of the assessment organization during five consecutive days.

**Figure 3:**
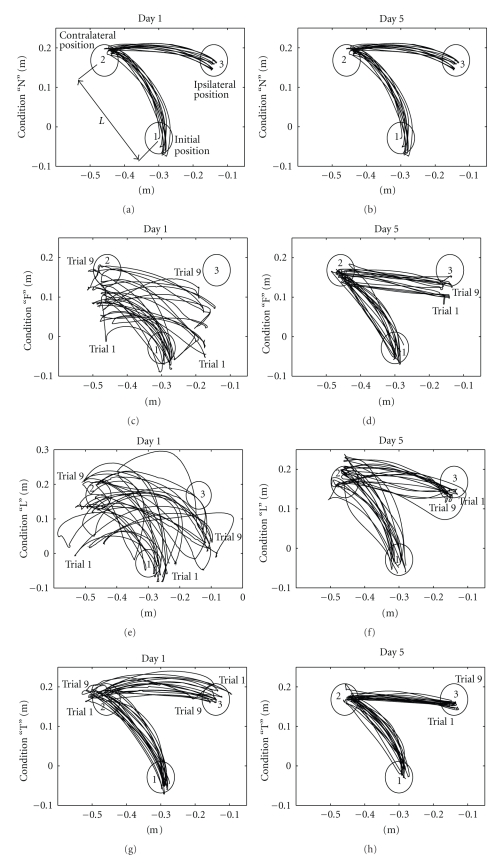
Original trajectories of one representative subject (9 trials) for the N, F, L, and T conditions on Days 1 and 5. The axes represent horizontal and vertical distances in meters. For each trial, the whole movement, comprised of four sequences, is plotted. The notations are 1—initial hand position, 2—circle at the contralateral position, and 3—circle at the ipsilateral position.

The subjects' altered vision was created by positioning the camera in front (F) of the subject, providing a mirror-like view, lateral (L) view from the right side, and top (T) view where the camera was recording from the position above the table. The camera projected the image from these viewpoints to the goggles (Figure 1, insert). The experimental procedure was the following. The reaching (manipulation) task that we studied comprised the following four sequence of activities: (1) move the hand from the initial position (1—Figure 1) to the small object placed at the contralateral target (2—Figure 1), (2) grasp a small cylinder (D = 2 cm, H = 1 cm) placed on the contralateral target, and move it to the ipsilateral target (3, Figure 1), (3) return the object back to the contralateral target (2) and release object, and (4) return the hand to the initial position. The subjects were instructed to go through all four sequences even if they failed to grasp the object in sequence 2. This “fail to grasp” case was treated as unsuccessful in later analysis. Subjects were also instructed to stop between sequences for about 2 seconds to allow clear separation of sequences in later analysis.

The analysis followed the protocol depicted in Figure 2.

The recordings on Day 1 were used as the baseline assessment. The session comprised three 30-second trials under all conditions with the altered vision (F, L, and T) and normal vision. In each trial the subject was asked to repeat the task as many times as he could. In most cases the subject accomplished the task three times. This provided in average nine data sets for each condition.

Days 2, 3, and 4 (Figure 2) were allocated for training, which consisted of performing the task for 20 minutes under the F condition of altered vision. The decision to allow subjects to practice only in one condition was made to allow analysis of the effects of practice on the performance of the movement. In this way, the performance for movements that were practiced (F condition) could be compared with the performance of those that were not (L and T conditions). 

The final evaluation was on Day 5 with the same protocol as the one described for Day 1.

### 2.3. Data Acquisition

The kinematics of arm-end point during reach and grasp activities were recorded in the Human Performance Lab at the Center for Sensory-Motor Interaction, Aalborg University, using a motion capture system (ProReflex MCU240, Qualisys, SE) with six cameras mounted on the tripods and positioned around the workspace. Two markers were placed on the lateral and medial aspects of the wrist. The marker positions were acquired at 50 Hz using Qualisys Track Manager (Qualisys, SE) and then exported to Matlab. The 3D trajectory of the wrist joint was calculated as the mean of the two recorded marker trajectories and was then projected onto the plane coincident with the surface of the table to obtain the resulting 2D trajectory of the movement. The calculated signal was filtered using a second-order dual-pass Butterworth filter at a cutoff frequency of 10 Hz based on previously published literature [25, 26].

### 2.4. Data Analysis

We distinguished between performed and unsuccessful tasks when the subject failed to grasp the object. This unsuccessful case was termed “no pick-up” error, and the measure was the number of “no pick-up” errors. The evaluation of the successful trials comprised analysis of the following: (1) End-point Error—EE, (2) sequence parameters, and (3) time of execution.

1. *End-Point-Errors* (EEs). An end-point error (EE) was defined as the distance between the reference point (the center of the circle in the workspace) and the actual end point of the trajectory for sequences 1, 2, and 3. We distinguished between the contralateral 1 EE (end of sequence 1), ipsilateral EE (end of sequence 2), and contralateral 2 EE (end of sequence 3).
2. *Sequence Parameters*. Peak velocity (PV), acceleration phase duration (AD), and deceleration phase duration (DD) were computed for each of the four sequences. PV was defined as the highest point on the velocity profile. AD was defined as the time from the onset of sequence movement to the time of peak velocity. DD was defined as the time from the peak velocity to the end of the sequence movement. Sequence movement onset and the end of the sequence movement were defined as times when the velocity was higher or lower than 5% of the peak velocity, respectively.
3. *Time of Execution* (TE). TE was defined as the total duration of the complex four-sequence movement.

### 2.5. Statistical Analysis

A one-way repeated-measures analysis of variance (ANOVA) was used to assess differences in errors between the first and fifth day. Significant differences were determined by the Student-Newman, Keuls test for multiple comparisons. The outcomes were declared significant at *P* < .05.

## 3. Results

Figure 3 presents representative trajectories of one subject for all conditions on Days 1 and 5 on the left and right plots, respectively. 

Note that on Day 1, the trajectories were scattered within the workspace, especially for conditions F and L. Furthermore, the end points of the individual sequences often ended up outside of the reference circles. On Day 5, the trajectories were more consistent, and the end points accumulated within or in close proximity to the reference circles. 

**Figure 4:**
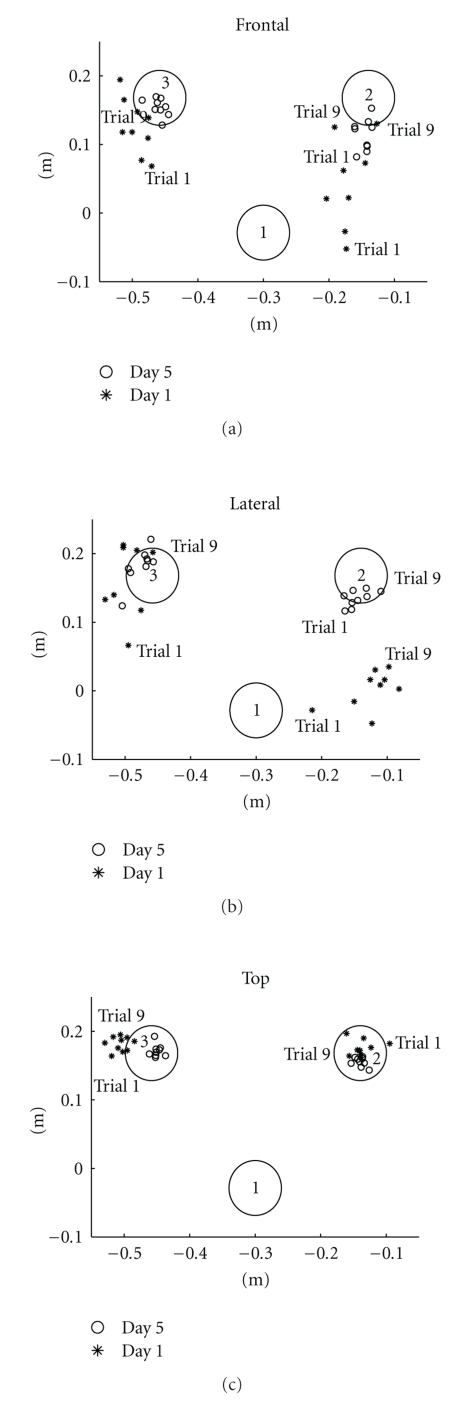
End points of trajectories under the F, L, and T experimental conditions for one representative subject on Day 1 (star) and Day 5 (full circle). The three circles with 8 cm diameter correspond to the initial hand position (circle 1) and the contralateral (circle 2) and ipsilateral (circle 3) circles in the workspace. End points of the trajectories (for 9 trials) are plotted for sequence 1 and sequence 2.

The latter is also demonstrated in Figure 4, which shows only the end points of the sequence trajectories on Days 1 and 5. On Day 5, the end-point clusters were less spread out, and their centers converged more towards the reference positions. 

**Figure 5:**
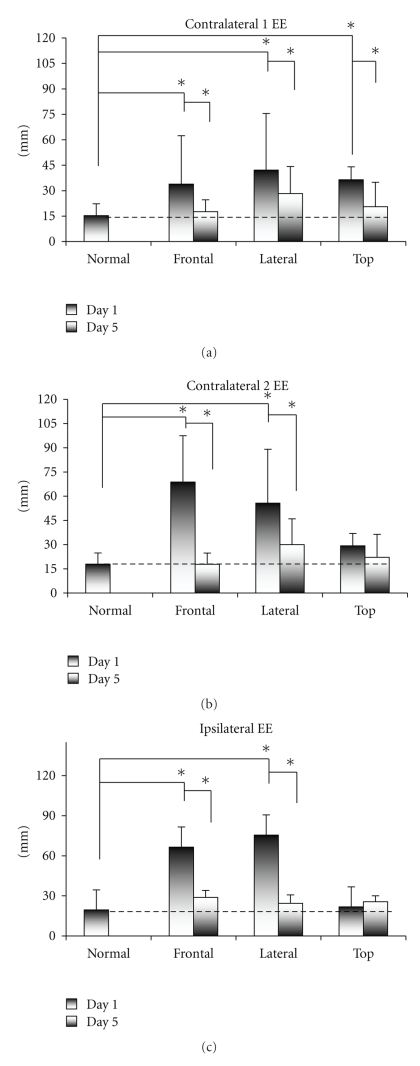
End-point errors for the four experimental conditions (N, F, L, and T) on Day 1 and Day 5. The errors are expressed in millimeters and represent group results. Significant differences between Day 1 and Day 5 and between N and F, L, and T conditions (*P* < .05) are illustrated (*). Condition N is presented with one result as the data were not changed from Day 1 to Day 5.

Figure 5 presents the overall end-point errors (EEs) for the four experimental conditions for both Day 1 and Day 5. The plots (from top to bottom) show the statistical data for contralateral 1 EE, contralateral 2 EE, and ipsilateral EE, respectively. These results show significant differences between Day 1 and Day 5 (for F, L, and T) and between the N and F, L, and T conditions (*P* < .05). The contralateral 1 EE was higher before the training sessions than after, and this difference was statistically significant for the F (F(2,29) = 7.83, *P* < .03), L (F(2,29) = 6.73, *P* < .02), and T (F(2,29) = 4.09, *P* < .04) conditions. In addition, contralateral 1 EE was greater for the F, L, and T conditions on Day 1 than the EE for the N condition, and this difference was statistically significant for the F, L, and T conditions (F(2,29) = 7.28, *P* < .03); (F(2,29) = 5.43, *P* < .01); (F(2,29) = 3.64, *P* < .05). On Day 5, the errors for the F, L, and T conditions became comparable with those for the N condition.

We show one result for the N-condition because there was no difference in the recordings between Days 1 and 5. There was no Negative Aftereffect [23]. 

The contralateral 2 EE was higher before the training sessions than after, and this difference was statistically significant for the F (F(2,29) = 8.24, *P* < .03) and L (F(2,29) = 6.23, *P* < .05) conditions. In addition, contralateral 2 EE for the F, L, and T conditions was greater on Day 1 than the EE for the N condition, and this difference was statistically significant for the F and T conditions (F(2,29) = 8.12, *P* < .02; (F(2,29) = 7.46, *P* < .05). On Day 5, the errors for the F, L, and T conditions became comparable with those for the N condition.

The ipsilateral EE was also higher before the training sessions than after, and this difference was statistically significant for the F (F(2,29) = 7.12, *P* < .03) and L (F(2,29) = 8.78, *P* < .05) conditions. In addition, the ipsilateral EE for the F, L, and T conditions was greater on Day 1 compared with the EE for the N condition, and this difference was statistically significant for the F and L conditions (F(2,29) = 7.68, *P* < .03; (F(2,29) = 8.26, *P* < .04). On Day 5, the errors for the F, L, and T conditions became similar to those for the N condition.

Table 1 summarizes the incidence of failed pick-up of the object for all conditions on Days 1 and 5. On Day 1, the no pick-up number was 47 out of 215 trials, whereas on Day 5 this occurred only 6 times out of 245 trials. 

**Figure 6:**
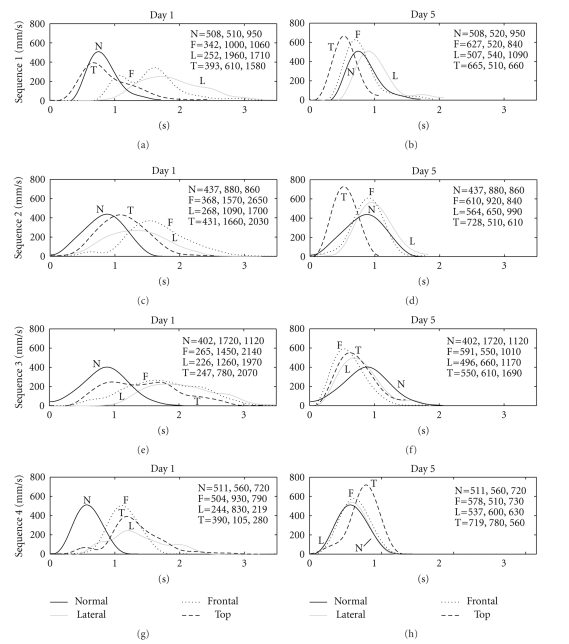
Velocity profiles of one representative subject for the four experimental conditions (N, F, L, and T) on Day 1 (left plots) and Day 5 (right plots). The four rows correspond to the four sequences of the movement. PV (m/s), AD (s), and DD (s) values are included for each sequence and each condition.

Figure 6 depicts the velocity profiles for one representative subject on Day 1 and on Day 5 under the four experimental conditions. Note that the velocities on Day 1 had unusual shapes (e.g., wavy and/or multimodal profiles). On Day 5, the velocities had near symmetrical bell-shaped profiles typical of normal reaching movements. Table 2 summarizes the movement parameters for the whole group under the four experimental conditions for Days 1 and 5. PV was higher for Day 5 than for Day 1 for all conditions and all movement sequences.

For all conditions with altered vision, there was an obvious decrease in AD and DD on Day 5 compared with Day 1 (Table 2). On Day 5, these values became comparable with those for the N condition. For all conditions with altered vision, there was an evident decrease in TE on Day 5 compared with Day 1 (Table 2). On Day 5, the value of TE for the F, T, and L conditions became comparable with those for the N condition.

## 4. Discussion

### 4.1. Poor Performance under Altered Vision

Figure 3 demonstrates that the altered visual input significantly affected the performance on Day 1. Subjects showed very poor performance for the F and L conditions and the trajectories scattered, covering almost the entire workspace. The poor performance for the F and L conditions observed qualitatively from the trajectory traces (Figure 3) is consistent with the high EE values for these conditions compared with condition N, as illustrated in Figure 5. The largest values for the contralateral 1 EE and ipsilateral EE were observed in the L condition, whereas the contralateral 2 EE reached a maximum value in the F condition. This suggests that altered vision in the L condition had the greatest effect on sequences 1 and 2, whereas altered vision in the F condition mostly affected sequence 3 of the movement. 

These observations are consistent with the scattered end points shown in Figure 4. On Day 1, the end points of the trajectories for the F and L conditions were scattered over a large area of the workspace outside of the reference circles. On the other hand, the end points for condition T were clustered together, within or very close to the reference circles, which suggests a lower level of dissociation of visual input and proprioception compared with the F and L conditions. This observation was also confirmed by the questionnaire that subjects filled out after the experiment. The subjects ranked F condition as the most difficult, followed by the L and T conditions.

The high trajectory errors and end-point variability on Day 1 were accompanied by an increase in AD, DD, and TE and a decrease in PV for the F, L, and T conditions, as presented in Table 2. Note that the time of execution (TE) was shorter in N (7, 26 s) than in the F (9, 77 s), L (9,17 s), and T (9,02 s) conditions. When visual input is altered, subjects often use a strategy of slowing down the movement to ensure accurate reach and grasp [26]. When the visual feedback of movement is presented on a screen, the movement accuracy decreases and the movement time increases [27].

Movement time tends to increase when visual feedback is reduced (e.g., when the vision was occluded at four different latencies from onset of the reach, as shown by Winges et al. [9]), mostly due to a longer deceleration phase of the movement caused by a slower approach to the object [5–9]. Indeed, the deceleration phase duration (DD) values for the group (Table 2) were longer in the F (1.1, 1.3, 1.4, 1.1 s) and L (1.0, 1.1, 1.0, and 0.94 s) conditions compared to N (0.81, 0.85, 0.83, and 0.84 s) condition for all four sequences of the movement. An increase in the duration of the deceleration phase (DD) of the reach was also found when visual feedback was blocked during the entire movement or only during the initial part of the movement [5, 9–11], when vision of the hand was blocked [5–7, 12, 13], or when monocular vision was used [8, 14, 15]. Note also that the acceleration phase (AD) for the F and L conditions lasts longer than in the N condition. This is presented in Figure 6 for a representative subject and in Table 2 for the whole group.

The presented data further extend the findings of Van Opstal and Van Gisbergen [28]; Sivak and MacKenzie [29]; Chieffi and Gentilucci [30]; and Berthier et al. [6] by showing that altered vision leads to a decrease in the peak velocity for all conditions and all sequences and that the bell-shaped velocity profile is absent in the F and L conditions, as illustrated for a representative subject in Figure 6. These changes in the nature of the velocity profile on Day 1 with respect to unaltered vision were accompanied by a greater total duration of each sequence (3.5 s on Day 1 compared with 1.5–2.0 s on Day 5). These observations are consistent with those for the whole group, as shown in Table 2.

### 4.2. Fast Learning

Subjects' performance even increased across trials on Day 1 as shown in Figure 3. For the representative subject presented in Figure 3, contralateral 1 EE decreased from 54 mm for trial 1 to 46 mm for trial 9, contralateral 2 EE decreased from 86 mm for trial 1 to 67 mm for trial 9, and ipsilateral EE decreased from 96 mm for trial 1 to 81 mm for trial 9 (F condition). This suggests that fast learning (recalibration) occurred but that adaptation remained incomplete. The performance improved on Day 5 due to trial-by-trial learning on Days 2, 3, and 4. The sensory systems rapidly adapted to the disrupted visual feedback, returning perception and performance to near normal. This finding might suggest that two correction mechanisms are involved in trajectory amendment: an initial mechanism that produces a quick but approximate reduction of spatial error between terminal hand position and target position and a complementary mechanism that leads to a progressive refinement and optimization of the trajectory through practice [25].

On Day 5, EE and no pick-up number decreased for all altered vision conditions as presented in Figure 5 and Table 1. This decrease translates to an increased ability of the subject to control the hand trajectory during the reach to grasp task. The improved performance was found for all views, although the training was performed for only one altered vision condition (F-view). The values became comparable with the values that are typical for the N condition in all conditions. This suggests that dissociation of the proprioception and vision introduced with the goggles was minimized with short learning and that recalibration occurred even for the views that were not practiced. This follows the results presented by Baraduc and Wolpert [16] who reported that the brain quickly adapted to a variety of distortions of visual feedback of the hand when reaching for targets, including rotations and lateral shifts in the field of view, by adjusting hand movements. 

Note that on Day 5 the variability of the trajectory end points decreased (full circles on Figure 4). The trajectory end points were clustered within the more narrow area; for sequence 1, almost all trajectory end points were inside the circle, whereas for sequence 2 some of the end points were still outside the circle. Improvement of terminal accuracy was associated with a change in kinematics parameters. The duration of the acceleration and deceleration periods and the time of execution decreased during the final trials. After practice, the time of execution decreased for the F (from 9.77 s on Day 1 to 8.24 s on Day 5) and L conditions (from 9.17 s on Day 1 to 7.82 s on Day 5). Concomitant with the reduction in the time of execution, there was a progressive increase of the peak velocity. The velocity profiles for the F, L, and T conditions became comparable with those for the N condition (bell shaped profile [3]), as shown in Figure 6. 

A similar pattern (decrease in the trajectory errors and increase in peak velocity from Day 1 to Day 5) was obtained in the F and L conditions in contrast to the T condition. This result suggests that although a general learning occurred, it was not at the same level for all views.

### 4.3. Visuomotor Skill Acquisition or Perceptual Recalibration?

Video-controlled reaching tasks represent a complex and original visuomotor situation because there is a discrepancy between the working and visual spaces, implying more elaborate processing of spatial information [25]. 

We analyzed subjects' performance on the first and the fifth days of goggle use to assess their ability to learn reaching and grasping with an altered visual input. We tested how the CNS deals with imposed artificial visual feedback compared with normal visually guided reaching. On Day 1, the altered vision resulted in worse performance than normal vision. However, by Day 5, the sensory systems had adapted to the discrepancy, returning perception and performance to near normal. 

One of the suggestions of these results is that this adaptation consists of “recalibrating” the transformation between the visual and proprioceptive perception of spatial location [24]. Perceptual recalibration appears to involve a global topological realignment, in the sense that alterations within a trained region of space are generalized to other untrained regions [31]. This is supported by our results showing an improved performance on Day 5 for the L condition, although this condition was not used for training. 

We do not assume that perceptual recalibration (a coordinative remapping between different perceptual representations such as vision and proprioception) and visual-motor skill acquisition (a task-dependent adjustment of the motor response to compensate for a manipulation of the working environment) are mutually exclusive [31–34]. On the contrary, we hypothesize that both occur; yet it is difficult to estimate the relative contribution of each of them over the course of the adaptation period. 

When visuomotor discrepancies occur, feedback that is perceived to be coincident with the limb is registered as an internal error, leading to the induction of a perceptual recalibration. Feedback that is not perceived to be physically coincident with the limb is registered as an external error, leading to the reduction of error during exposure [35]. It is also possible that the perception of the error as internal or external in origin might lead the subject to rely preferentially on either egocentric or allocentric cues for the guidance of movement [35]. Studies have shown that there is a functional interaction between the two frames of reference and that this interaction can be affected by experimental conditions [36, 37].

Our results suggest that the difference between altered vision tasks and normal visually guided reaching leads to an adaptation in the form of perceptual recalibration, where proprioception is calibrated in terms of the visual system. If the adaptation is expected to take the guise of a more cognitive, problem-solving process, we can refer to this as the visual-motor skill acquisition. Future studies are warranted to further explore this issue. The ability to predict with some confidence which of these two types of adaptation a peripheral manipulation would allow for a prediction of whether significant improvement is likely to occur on training, how persistent the adaptation will be, and whether it will result in Aftereffects [38].

One of the envisioned applications of the results of this study is for rehabilitation of stroke patients. In stroke patients, a dissociation of proprioception and vision is caused due to the impaired sensory-motor systems. The accepted approach for effective therapy suggests intensive repetitive exercise, being possibly augmented with assistive systems such as functional electrical stimulation [39] or assistant robots [40]. These therapies allow patients to train performing functional movements and learn new strategies of optimal use of preserved sensory-motor mechanisms. This training could be understood as the process of recalibration of the natural control system. The results of this study show that in healthy individuals, this recalibration is fast and effective.

The other application that is envisioned relates to the inclusion of cognitive vision in the control loop for transradial prosthesis [41, 42]. In this case, the camera is integrated into artificial hand; therefore, the camera moves and generates the altered visual input, which the controller needs to adapt to.

## 5. Conclusion

In this paper, we presented an effective, yet simple new tool for altering visual input when studying motor coordination of reaching during the reach to grasp task. The results show that this alteration of visual input can be graded and, hence, allow for the study of different concepts of learning of the movement. 

This study partly confirms the negative aftereffect acting after perceptual recalibration due to altered visual input. Namely, the results confirm that the learning of a new skill and perceptual recalibration acted with different proportion during the adaptation period. However, we need to restate that the learning of a new task has not disrupted the previous skills (normal condition); therefore, suggesting no negative aftereffect.

## Figures and Tables

**Table 1:** Number of “no pick-up” trials and the maximum errors 1EE, EE, and 2EE averaged over the group for *N*, *F*, *L*, and *T* conditions. The grey fields show the worst performance: the *L* and *T* altered vision conditions result with much bigger errors compared with the *T* condition errors.

Condition	Number of “no pick up”		Max error 1EE [mm]		Max error EE [mm]		Max error 2EE [mm]	
	Day 1	Day 5	Day 1	Day 5	Day 1	Day 5	Day 1	Day 5
Normal	0 out of 80 (**0%**)	0 out of 80 (**0%**)	15 ± 6		19 ± 14		19 ± 8	
Frontal	13 out of 70 (**18.6%**)	0 out of 80 (**0%**)	32 ± 29	16 ± 8	63 ± 12	**30 ± 15**	**75 ± 16**	23 ± 7
Lateral	24 out of 65 (**36.9%**)	4 out of 75 (5.3%)	**38 ± 34**	**28 ± 15**	**68 ± 30**	22 ± 7	66 ± 14	**28 ± 5**
Top	10 out of 80 (**12.5%**)	2 out of 90 (2.2%)	28 ± 6	22 ± 11	16 ± 11	22 ± 3	30 ± 6	22 ± 14

**Table 2:** Movement parameters (group results). Sequence parameters (peak velocity-PV, acceleration phase duration-AD, and deceleration phase duration-DD) and time of execution (TE) are given for the normal, frontal, lateral, and top experimental conditions. The numbers (1, 2, 3, and 4) below the parameters correspond to the sequences 1, 2, 3, and 4 of the movement.

	Sequence parameters (Mean ± SD)													
		PV [m/s]				AD [s]				DD [s]				TE [s]
Condition	Day	1	2	3	4	1	2	3	4	1	2	3	4	

Normal	1/5	0.55 ± 0.01	0.54 ± 0.09	0.47 ± 0.04	0.48 ± 0.08	0.54 ± 0.13	0.71 ± 0.21	0.90 ± 0.15	0.62 ± 0.15	0.81 ± 0.13	0.85 ± 0.22	0.83 ± 0.10	0.84 ± 0.14	7.26 ± 1.24
Frontal	1	0.44 ± 0.14	0.42 ± 0.06	0.39 ± 0.13	0.38 ± 0.05	0.61 ± 0.10	0.91 ± 0.33	1.03 ± 0.38	0.70 ± 0.08	1.10 ± 0.47	1.31 ± 0.29	1.42 ± 0.53	1.09 ± 0.18	9.77 ± 2.55
	5	0.52 ± 0.11	0.55 ± 0.07	0.51 ± 0.07	0.52 ± 0.10	0.52 ± 0.07	0.68 ± 0.12	0.62 ± 0.06	0.61 ± 0.07	0.75 ± 0.09	0.96 ± 0.19	0.91 ± 0.19	0.68 ± 0.20	8.24 ± 1.46
Lateral	1	0.42 ± 0.12	0.41 ± 0.14	0.42 ± 0.15	0.41 ± 0.07	0.79 ± 0.23	0.91 ± 0.17	0.98 ± 0.11	0.83 ± 0.02	1.00 ± 0.38	1.07 ± 0.41	1.04 ± 0.30	0.94 ± 0.37	9.17 ± 1.90
	5	0.51 ± 0.10	0.57 ± 80	0.54 ± 0.24	0.55 ± 0.12	0.55 ± 0.04	0.64 ± 0.05	0.67 ± 0.09	0.57 ± 0.09	0.85 ± 0.18	0.93 ± 0.17	1.02 ± 0.16	0.82 ± 0.20	7.82 ± 0.94
Top	1	0.48 ± 0.08	0.48 ± 0.07	0.42 ± 0.13	0.41 ± 0.03	0.53 ± 0.04	0.94 ± 0.36	0.76 ± 0.15	0.76 ± 0.07	0.89 ± 0.44	1.08 ± 0.22	1.39 ± 0.42	1.46 ± 0.59	9.02 ± 1.81
	5	0.57 ± 0.07	0.71 ± 0.01	0.58 ± 0.02	0.67 ± 0.02	0.70 ± 0.18	0.70 ± 0.16	0.74 ± 0.18	0.75 ± 0.16	0.82 ± 0.19	1.07 ± 0.20	1.14 ± 0.25	0.68 ± 0.08	8.79 ± 0.96

## References

[1] Jeannerod M, Long J, Baddeey A (1981). Intersegmental coordination during reaching at natural visual objects. *Attention and Performance IX*.

[2] Jeannerod M (1999). Visuomotor channels: their integration in goal-directed prehension-. *Human Movement Science*.

[3] Jeannerod M (1984). The timing of natural prehension movements. *Journal of Motor Behavior*.

[4] Jakobson LS, Goodale MA (1991). Factors affecting higher-order movement planning: a kinematic analysis of human prehension. *Experimental Brain Research*.

[5] Schettino LF, Adamovich SV, Poizner H (2003). Effects of object shape and visual feedback on hand configuration during grasping. *Experimental Brain Research*.

[6] Berthier NE, Clifton RK, Gullapalli V, McCall DD, Robin DJ (1996). Visual information and object size in the control of reaching. *Journal of Motor Behavior*.

[7] Connolly JD, Goodale MA (1999). The role of visual feedback of hand position in the control of manual prehension. *Experimental Brain Research*.

[8] Watt SJ, Bradshaw MF (2000). Binocular cues are important in controlling the grasp but not the reach in natural prehension movements. *Neuropsychologia*.

[9] Winges SA, Weber DJ, Santello M (2003). The role of vision on hand preshaping during reach to grasp. *Experimental Brain Research*.

[10] Jackson SR, Jackson GM, Rosicky J (1995). Are non-relevant objects represented in working memory? The effect of non-target objects on reach and grasp kinematics. *Experimental Brain Research*.

[11] Schettino LF, Adamovich SV, Hening W, Tunik E, Sage J, Poizner H (2006). Hand preshaping in Parkinson’s disease: effects of visual feedback and medication state. *Experimental Brain Research*.

[12] Gentilucci M, Toni I, Chieffi S, Pavesi G (1994). The role of proprioception in the control of prehension movements: a kinematic study in a peripherally deafferented patient and in normal subjects. *Experimental Brain Research*.

[13] Churchill A, Hopkins B, Rönnqvist L, Vogt S (2000). Vision of the hand and environmental context in human prehension. *Experimental Brain Research*.

[14] Servos P, Goodale MA, Jakobson LS (1992). The role of binocular vision in prehension: a kinematic analysis. *Vision Research*.

[15] Jackson SR, Jones CA, Newport R, Pritchard C (1997). A kinematic analysis of goal-directed prehension movements executed under binocular, monocular, and memory-guided viewing conditions. *Visual Cognition*.

[16] Baraduc P, Wolpert DM (2002). Adaptation to a visuomotor shift depends on the starting posture. *Journal of Neurophysiology*.

[17] Yamamoto K, Hoffman DS, Strick PL (2006). Rapid and long-lasting plasticity of input-output mapping. *Journal of Neurophysiology*.

[18] Krakauer JW, Ghez C, Ghilardi MF (2005). Adaptation to visuomotor transformations: consolidation, interference, and forgetting. *Journal of Neuroscience*.

[19] Goodbody SJ, Wolpert DM (1998). Temporal and amplitude generalization in motor learning. *Journal of Neurophysiology*.

[20] Rossetti Y, Rode G, Boisson D (1995). Implicit processing of somaesthetic information: a dissociation between where and how?. *NeuroReport*.

[21] Kammers MPM, van der Ham IJM, Dijkerman HC (2006). Dissociating body representations in healthy individuals: differential effects of a kinaesthetic illusion on perception and action. *Neuropsychologia*.

[22] Van Beers RJ, Sittig AC, Gon JJ (1999). Integration of proprioceptive and visual position-information: an experimentally supported model. *Journal of Neurophysiology*.

[23] Kaernbach C, Munka L, Cunningham D, Würtz R, Lappe M (2002). Visuomotor adaptation: dependency on motion trajectory. *Dynamic Perception*.

[24] Bedford FL (1999). Keeping perception accurate. *Trends in Cognitive Sciences*.

[25] Pennel I, Coello Y, Orliaguet J-P (2002). Frame of reference and adaptation to directional bias in a video-controlled reaching task. *Ergonomics*.

[26] Germain R, Boy F, Orliaguet JP, Coello Y (2004). Visual and motor constraints on trajectory planning in pointing movements. *Neuroscience Letters*.

[27] Ferrel C, Leifflen D, Orliaguet J-P, Coello Y (2000). Pointing movement visually controlled through a video display: adaptation to scale change. *Ergonomics*.

[28] Van Opstal AJ, Van Gisbergen JAM (1987). Skewness of saccadic velocity profiles: a unifying parameter for normal and slow saccades. *Vision Research*.

[29] Sivak B, MacKenzie CL (1990). Integration of visual information and motor output in reaching and grasping: the contributions of peripheral and central vision. *Neuropsychologia*.

[30] Chieffi S, Gentilucci M (1993). Coordination between the transport and the grasp components during prehension movements. *Experimental Brain Research*.

[31] Bedford FL (1993). Perceptual and cognitive spatial learning. *Journal of Experimental Psychology*.

[32] Lackner JR, Dizio P (1994). Rapid adaptation to Coriolis force perturbations of arm trajectory. *Journal of Neurophysiology*.

[33] Martin TA, Keating JG, Goodkin HP, Bastian AJ, Thach WT (1996). Throwing while looking through prisms II. Specificity and storage of multiple gaze-throw calibrations. *Brain*.

[34] Redding GM, Wallace B (1996). Adaptive spatial alignment and strategic perceptual-motor control. *Journal of Experimental Psychology*.

[35] Clower DM, Boussaoud D (2000). Selective use of perceptual recalibration versus visuomotor skill acquisition. *Journal of Neurophysiology*.

[36] Gentilucci M, Chieffi S, Daprati E, Saetti MC, Toni I (1996). Visual illusion and action. *Neuropsychologia*.

[37] Gentilucci M, Daprati E, Gangitano M, Toni I (1997). Eye position tunes the contribution of allocentric and egocentric information to target localization in human goal-directed arm movements. *Neuroscience Letters*.

[38] Welch RB, Sampanes AC (2008). Adapting to virtual environments: visual-motor skill acquisition versus perceptual recalibration. *Displays*.

[39] Popović MB, 30. Popović DB, Sinkjær T, Stefanović A, Schwirtlich L (2003). Clinical evaluation of functional electrical therapy in acute hemiplegic subjects. *Journal of Rehabilitation Research and Development*.

[40] Huang VS, Krakauer JW (2009). Robotic neurorehabilitation: a computational motor learning perspective. *Journal of NeuroEngineering and Rehabilitation*.

[41] Klisić Dj, Kostić M, Došen S, Popović DB (2009). Control of prehension for the transradial prosthesis: natural-like image recognition system. *Journal of Automatic Control *.

[42] Došen S, Popović DB Transradial prosthesis: artificial vision for control of prehension.

